# Prevalence and Description of High‐Grade Intraepithelial Lesions of Cervical Mucosa Cells in a Screening Programme for Cervical Cancer in Isère, France

**DOI:** 10.1002/cam4.70259

**Published:** 2024-10-14

**Authors:** Christian Balamou, Karine Zysman, Cécile Olicard, Anne Garnier, Arnaud Seigneurin

**Affiliations:** ^1^ Université Grenoble Alpes Grenoble France; ^2^ Translational Innovation in Medicine and Complexity/Recherche Translationnelle et Innovation en Médecine et Complexité‐UMR 5525‐Laboratoire1043049 Domaine de la Merci La Tronche France; ^3^ Centre Régional de Coordination des Dépistages des Cancers en Auvergne‐Rhône‐Alpes Bourg‐en‐Bresse France; ^4^ Centre Régional de Coordination des Dépistages des Cancers en Auvergne‐Rhône‐Alpes Meylan France; ^5^ Registre du Cancer de l'Isère Centre Hospitalier Universitaire de Grenoble Grenoble Cedex 9 France

**Keywords:** cervical cancer screening, cervical pap smear, high‐grade intraepithelial lesions, HPV, prevalence, uterine cervical neoplasms

## Abstract

**Objective:**

This study aims to analyse high‐grade intraepithelial lesions (LIEHG) observed in a screening programme from 2010 to 2018 to more accurately describe them and formulate recommendations for best practices in the context of screening evolution following the introduction of an HPV test in primary cervical cancer screening in 2020.

**Methods:**

This study included 305,940 asymptomatic women aged 25–65 years. The eligible population was invited to undergo a screening cervico‐uterine‐smear every 3 years. If this smear was normal, the woman received a new invitation. In the case of a positive screen, the patient underwent further diagnostic procedures, such as colposcopy and biopsy to confirm the diagnosis. Only those diagnosed with LIEHG and above proceeded to treatment. The diagnoses associated with LIEHG were those related to the WHO Classification of tumours of the uterine cervix.

**Results:**

Positive smears led to the diagnosis of 3230 LIEHG. The prevalence of LIEHG in the screened population was 0.4%. The LIEHG distribution varied significantly according to the age of the women. The probability of diagnosing LIEHG in young women was 12.2% at 25–29 years. This probability decreased by half after 50 years. We observed that the type of smear was significantly associated with LIEHG detection. The positive predictive value for diagnosing LIEHG was 70.3% for high‐grade squamous intraepithelial lesion (HSIL) smears and 35.1% for atypical squamous cells cannot exclude HSIL (ASC‐H) smears.

**Conclusion:**

In the study population, the prevalence of LIEHG was high in women under 35 years. Low‐grade smears were correlated with the risk of LIEHG and should prompt screening facilities to allocate resources to ensure active follow‐up of LSIL and ASC‐US smears. Adherence to cytological screening recommendations was associated with a reduced risk of LIEHG in multivariate analysis.

## Graphical abstract

At the time of the data extraction 732,203 cervico‐uterine‐smears had been performed on 305,940 women. The number of first positive smear observed in each woman was retained in the analysis: 31,097 cases. These positive smears led to the diagnosis of 3,230 LIEHG.

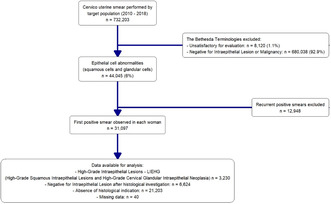

## 1 Introduction

Cervical cancer is the fourth most common cancer and the fourth cause of cancer death in women worldwide, with an estimated 660,000 new cases and 350,000 deaths globally in 2022 [1]. Its development has a strong etiological link with cervical mucosa cell infection by high‐risk human papillomavirus (HR‐HPV) types, in particular HPV16 and HPV18, even though a recent study by Pruski et al. indicated that HPV type 18 is no longer as common [2, 3, 4, 5, 6]. Other important cofactors include certain sexually transmissible infections, smoking, multiple sexual partners, age at first exposure and long‐term use of oral contraceptives [2, 7, 8, 9]. Cervical cancer is considered nearly completely preventable because of highly effective primary (HPV vaccine) and secondary screening prevention measures. After 13 years of experimentation based on pilot specifications [10, 11], in 2018, the French National Cervical Cancer Screening Program (FNCCSP) was initiated to reduce the incidence and mortality of this cancer [12]. Studies report that incidence and mortality reduction strategies for cervical cancer must combine actions on HPV vaccination for men and women, increasing screening coverage and efficient follow‐up of women with a positive screen for identification and early management of high‐grade intraepithelial lesions (LIEHG) [13]. In their study on HPV vaccine impact and effectiveness, Rosenblum et al. reported a 90% reduction in cervical HPV infections among vaccinated women [4]. Other authors also indicate that vaccinated populations experience fewer precancerous lesions, a decreased risk of recurrence after local excision treatment and an increased chance of HPV remission [14, 15, 16].

This study aims to analyse LIEHG observed in FNCCSP from 2010 to 2018 to more accurately describe them and formulate recommendations for best practices in the context of FNCCSP evolution following the introduction in 2020 of HPV tests as part of primary cervical cancer screening for women aged 30 to 65 years [17].

## 2 Materials and Methods

### 2.1 Study Design and Setting

We conducted a cross‐sectional retrospective analytical and descriptive study. We reported our results using the Strengthening the Reporting of Observational Studies in Epidemiology guidelines (STROBE) [18]. This study included all women living in Isère, a French department of the Auvergne‐Rhône‐Alpes region, between January 1, 2010 and December 31, 2018.

### 2.2 Screening Programme

At the time of data collection, a screening campaign was organised by the Isère anticancer departmental office [19] according to the FNCCSP pilot programme [11]. The target population was asymptomatic women aged 25–65 years, with no risk factors other than their age. The eligible population was invited every 3 years to undergo screening by conventional cervico‐uterine smear. In 2018, the more efficient liquid‐based Pap smear [20] was introduced into the screening programme [12]. If the result of this procedure was normal, the woman received a new invitation. In the case of a positive screen, the patient underwent further diagnostic procedures, such as colposcopy and biopsy, to confirm the diagnosis. Only those diagnosed with LIEHG and above proceeded to treatment. The smear sample was analysed by the Department of Anatomical Pathology or by certified (NF EN ISO 15189) medical laboratories [21].

### 2.3 Data Sources and Participants

The data analysed were extracted on 31 May 2023 from the Isère management database. This database was regularly enriched by socio‐demographic data, diagnostic data and follow‐up data provided by screening partners (health insurance plans, medical information services, gynaecologists, midwives, colposcopists, surgeons and general physicians). For data extraction from the database, we used an SQL query.

### 2.4 Variables

Smear collection: Screening cervico‐uterine‐smears were performed by nearby gynaecologists, midwives or general physicians. Abnormal or positive screening tests were described according to Bethesda terminology: low‐grade squamous intraepithelial lesion (LSIL), high‐grade squamous intraepithelial lesion (HSIL), atypical squamous cells of undetermined significance (ASC‐US), atypical squamous cells—cannot exclude HSIL (ASC‐H), squamous cell carcinoma, atypical glandular cells (not otherwise specified—NOS), atypical glandular cells (favour neoplastic), endocervical adenocarcinoma in situ, adenocarcinoma (endocervical, endometrial, extrauterine and NOS) and other malignant neoplasms [22]. LIEHG were described according to the WHO Classification of Tumours of the Uterine Cervix: high‐grade squamous intraepithelial lesions (cervical intraepithelial neoplasia, grade 2—CIN 2; cervical intraepithelial neoplasia, grade 3—CIN 3; moderate squamous dysplasia; severe squamous dysplasia; squamous carcinoma in situ—CIS) and high‐grade cervical glandular intraepithelial neoplasia (or adenocarcinoma in situ—AIS) [23]. The age at the time of a positive test (25–29; 30–34, 35–39; 40–44; 45–49; 50–54, 55–59, ≥ 60 years old) was described. Number of previous screenings corresponded to the number of prior participation in a cervical cancer screening campaign (both opportunistic and organised) in Isère (0, 1, 2, 3, 4, 5 and more). The time (in months) taken to perform the positive test was 6 months and below, 1 year ±6 months, 2 years ±6 months, 3 years ±6 months and 3.5 years and more.

### 2.5 Statistical Analysis

Qualitative variables were described as frequencies and quantitative variables as means ± standard deviation or median. Prevalence estimates of LIEHG were calculated as the number of LIEHG divided by the number of all screening cervico‐uterine‐smears and by the number of positive screens. The positive predictive value (PPV) of LIEHG among positive cervico‐uterine‐smears was estimated: the true positives (TP) were those whose diagnostic procedure confirmed LIEHG. The false positives (FP) were those whose diagnostic procedure did not confirm LIEHG (PPV = TP/[TP + FP]). A chi‐squared test and Fisher's exact test were used to compare the different proportions. The relationship between LIEHG (yes vs. no) and the predictive factors was analysed in a multivariate logistic regression estimating the adjusted odds ratio (OR) and its 95% confidence intervals (CI). We used multivariate logistic regression analysis because it is suitable for studying binary variables and allows us to measure the strength of the association between the LIEHG variable and the explanatory variables. Akaike's information criterion determined the best model (with and without interaction). Analysis was performed with version 4.3.1 of R software. Statistical significance was set at *p* ≤ 0.05.

## 3 Results

**FIGURE 1:**
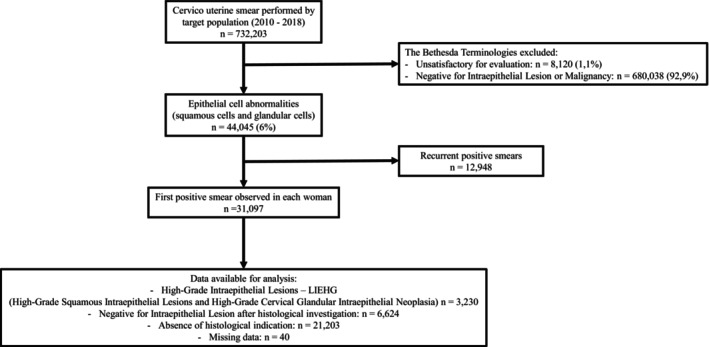
Flow diagram of the study workflow.

At the time of data extraction, 732,203 cervico‐uterine‐smears had been performed on 305,940 women. The number of positive smears was 44,045 (6.0%), and several positive smears were observed in some women (positive control smears as part of follow‐up). The first positive smear observed in each woman was retained in the analysis: 31,097 cases, which represents 4.2% of all smears. These positive smears led to the diagnosis of 3230 LIEHG (Figure 1: Flowchart).

### 3.1 Prevalence of LIEHG

In the study, the mean age at diagnosis of LIEHG was 36.8 (±9.0) years. The prevalence of LIEHG in the screened population was 0.4% (95% CI 0.4%–0.5%) (Table 1). The age‐based prevalence of LIEHG was 0.81% at 25–29 years, 0.71% at 30–34 years, 0.57% at 35–39 years, 0.47% at 40–44 years, 0.33% at 45–49 years, 0.17% at 50–54 years, 0.13% at 55–59 years and 0.09% at 60 years and older. The LIEHG prevalence distribution by year among positive smears shows a slight increase between 2013 and 2016 (Table 1). According to the type of LIEHG diagnosed in positive smears, the prevalence of high‐grade squamous intraepithelial lesions (CIN 2: 4.2%, CIN 3: 5.9% and CIS: 0.1%) is the highest compared to high‐grade cervical glandular intraepithelial neoplasia (0.1%). In 0.1% of cases, these two types of LIEHG coexist (Table 2). The 3230 LIEHG diagnosed comprised 57.0% CIN 3, 40.7% CIN 2, 0.5% CIS, 0.7% AIS, 0.8% simultaneous AIS and CIN2/3 and 0.3% LIEHG associated with other malignant neoplasms.

**TABLE 1:** Distribution and prevalence of high‐grade intraepithelial lesions among all screenings and first positive smear.

Characteristics	All screening y‐A: (n1/y)	Number of first positive smear n (%)	Number of LIEHG m (%)	Prevalence of LIEHG among all screening		Prevalence of LIEHG among first positive smear	
				% (m/y)	95% CI	% (m/n)	95% CI
Overall	732,203‐A: 6.0 (44,045/732,203)	31,097 (100)	3230 (100)	0.4% (3230/732,203)	0.4–0.5	10.4% (3230/31,097)	10.0–10.7
Age	40 (32–47)^a^/40.0 (±9.9)^b^	39 (31–47)^a^/39.4 (±10.1)^b^	35 (30–43)^a^/36.8 (±9.0)^b^				
Years screening							
2010	75,418‐A: 4.8 (3603/75,418)	3052 (9.8)	242 (7.5)	0.3% (242/75,418)	0.3–0.3	7.9% (242/3052)	7.0–9.0
2011	86,821‐A: 4.0 (3455/86,821)	2651 (8.5)	238 (7.4)	0.3% (238/86,821)	0.3–0.3	9.0% (238/2651)	7.9–10.1
2012	81,369‐A: 4.4 (3567/81,369)	2644 (8.5)	260 (8.0)	0.3% (260/81,369)	0.3–0.3	9.8% (260/2644)	8.7–11.0
2013	77,302‐A: 5.5 (4253/77,302)	3041 (9.8)	345 (10.7)	0.4% (345/77,302)	0.4–0.4	11.3% (345/3041)	10.2–12.5
2014	76,620‐A: 5.6 (4315/76,620)	3028 (9.8)	440 (13.6)	0.6% (440/76,620)	0.6–0.6	14.5% (440/3028)	13.3–15.8
2015	80,060‐A: 6.3 (5057/80,060)	3578 (11.5)	476 (14.7)	0.6% (476/80,060)	0.6–0.6	13.3% (476/3578)	12.2–14.5
2016	84,435‐A: 7.0 (5890/84,435)	3933 (12.7)	483 (15.0)	0.6% (483/84,435)	0.6–0.6	12.3% (483/3933)	11.3–13.3
2017	84,388‐A: 7.6 (6405/84,388)	4287 (13.8)	426 (13.2)	0.5% (426/84,388)	0.5–0.5	9.9% (426/4287)	9.1–10.9
2018	85,790‐A: 8.7 (7500/85,790)	4883 (15.7)	320 (9.9)	0.4% (320/85,790)	0.4–0.4	6.6% (320/4883)	5.9–7.3

Abbreviations: 95% CI, 95% confidence interval; A, ratio (in %) of all positive smears divided by the total number of smears in the modality (a woman can have several positive smears); m, total number of high‐grade intraepithelial lesions (LIEHG) in the modality; n, total number of positive smears in the modality (one positive smear per woman); n1, total number of all positive smears in the modality (a woman can have several positive smears); y, total smears in the modality.

^a^ Median (first quartile–third quartile).

^b^ Mean (±standard deviation).

**TABLE 2:** Distribution of high‐grade intraepithelial lesion types among different characteristics.

Characteristics	Number of first positive smear	Positive predictive value of LIEHG % (m/y)	Number of types of LIEHG						
			CIN 2 m (%)^c^	CIN 3 m (%)^c^	CIS m (%)^c^	AIS m (%)^c^	CIN 2/3 + AIS m (%)^c^	LIEHG and other tumours m (%)^c^	Overall LIEHG m (%)^c^
Overall	31,097^a^	3230^a^	4.2%^b^	5.9%^b^	0.1%^b^	0.1%^b^	0.1%^b^	0.0%^b^	10.4%^b^
			1313 (40.7)	1842 (57.0)	16 (0.5)	24 (0.7)	25 (0.8)	10 (0.3)	3230 (100)
Age									
25–29	6585	12.2% (804/6585)	353 (43.9)	435 (54.1)	5 (0.6)	6 (0.8)	5 (0.6)	—	804 (100)
30–34	5075	14.1% (714/5075)	286 (40.1)	418 (58.6)	3 (0.4)	3 (0.4)	3 (0.4)	1 (0.1)	714 (100)
35–39	4702	12.5% (586/4702)	219 (37.4)	350 (59.7)	3 (0.5)	5 (0.9)	7 (1.2)	2 (0.3)	586 (100)
40–44	4822	10.2% (494/4822)	183 (37.1)	295 (59.7)	3 (0.6)	5 (1.0)	5 (1.0)	3 (0.6)	494 (100)
45–49	4448	7.4% (330/4448)	156 (47.3)	168 (50.9)	1 (0.3)	1 (0.3)	2 (0.6)	2 (0.6)	330 (100)
50–54	2903	5.2% (150/2903)	59 (39.3)	85 (56.7)	1 (0.7)	1 (0.7)	3 (2.0)	1 (0.7)	150 (100)
55–59	1480	6.2% (92/1480)	36 (39.1)	53 (57.6)	—	3 (3.3)	—	—	92 (100)
60 and older	1082	5.5% (60/1082)	21 (35.0)	38 (63.3)	—	—	—	1 (1.7)	60 (100)
Number of previous screenings									
0	14,470	11.6% (1684/14,470)	639 (37.9)	1010 (60.0)	13 (0.8)	9 (0.5)	8 (0.5)	5 (0.3)	1684 (100)
1	8201	10.0% (819/8201)	372 (45.4)	427 (52.1)	1 (0.1)	8 (1.0)	8 (1.0)	3 (0.4)	819 (100)
2	4401	9.5% (420/4401)	164 (39.0)	245 (58.3)	2 (0.5)	5 (1.2)	2 (0.5)	2 (0.5)	420 (100)
3	2315	8.0% (185/2315)	78 (42.2)	100 (54.1)	—	1 (0.5)	6 (3.2)	—	185 (100)
4	986	8.9% (88/986)	42 (47.7)	44 (50.0)	—	1 (1.1)	1 (1.1)	—	88 (100)
5 and more	724	4.7% (34/724)	18 (52.9)	16 (47.1)	—	—	—	—	34 (100)
Time taken to perform the positive test (months)—Around the year									
First screening	14,470	11.6% (1684/14,470)	639 (37.9)	1010 (60.0)	13 (0.8)	9 (0.5)	8 (0.5)	5 (0.3)	1684 (100)
6 months and below	514	7.8% (40/514)	25 (62.5)	14 (35.0)	—	1 (2.5)	—	—	40 (100)
1 year ±6 months	4019	8.6% (346/4019)	158 (45.7)	178 (51.4)	1 (0.3)	3 (0.9)	5 (1.4)	1 (0.3)	346 (100)
2 years ±6 months	5324	9.5% (505/5324)	222 (44.0)	274 (54.2)	1 (0.2)	3 (0.6)	3 (0.6)	2 (0.4)	505 (100)
3 years ±6 months	3292	10.2% (335/3292)	150 (44.8)	176 (52.5)	—	4 (1.2)	5 (1.5)	—	335 (100)
3.5 years and more	3478	9.2% (320/3478)	119 (37.2)	190 (59.3)	1 (0.3)	4 (1.3)	4 (1.3)	2 (0.6)	320 (100)
Smear collectors									
General physicians	7463	10.1% (755/7463)	326 (43.2)	412 (54.6)	7 (0.9)	4 (0.5)	6 (0.8)	—	755 (100)
Gynaecologists	22,107	10.6% (2337/22,107)	931 (39.9)	1349 (57.7)	9 (0.4)	19 (0.8)	19 (0.8)	10 (0.4)	2337 (100)
Midwives	939	10.9% (102/939)	42 (41.2)	59 (57.8)	—	1 (1.0)	—	—	102 (100)
Others	30	6.7% (2/30)	—	2 (100)	—	—	—	—	2 (100)
Missing	558	34	14	20	—	—	—	—	34 (100)
Types of positive smears									
ASC‐US	18,087	4.7% (855/18,087)	392 (45.9)	455 (53.2)	1 (0.1)	2 (0.2)	3 (0.4)	2 (0.2)	855 (100)
LSIL	9666	9.7% (941/9666)	464 (49.3)	459 (48.8)	5 (0.5)	3 (0.3)	8 (0.9)	2 (0.2)	941 (100)
ASC‐H	1366	35.1% (480/1366)	203 (42.3)	271 (56.5)	3 (0.6)	2 (0.4)	1 (0.2)	—	480 (100)
HSIL	1249	70.3% (878/1249)	240 (27.3)	610 (69.5)	7 (0.8)	7 (0.8)	10 (1.1)	4 (0.5)	878 (100)
Squamous Carcinoma in situ	40	22.5% (9/40)	1 (11.1)	8 (88.9)	—	—	—	—	9 (100)
Squamous cell carcinoma	1	100% (1/1)	—	—	—	—	—	1 (100)	1 (100)
Atypical glandular cells	377	17.2% (65/377)	13 (20.0)	38 (58.5)	—	10 (15.4)	3 (4.6)	1 (1.5)	65 (100)
Atypical endometrial cells	290		—	—	—	—	—	—	—
Adenocarcinoma	21	4.8% (1/21)	—	1 (100)	—	—	—	—	1 (100)

Abbreviations: ASC‐H, atypical squamous cells cannot exclude HSIL; ASC‐US, atypical squamous cells of undetermined significance; HSIL, high‐grade squamous intraepithelial lesion; LIEHG, high‐grade intraepithelial lesions; LSIL, low‐grade squamous intraepithelial lesion; m, total number of LIEHG in the modality; y, total number of positive screenings in the modality.

^a^ Effective.

^b^ Prevalence (%) of types of LIEHG (m/31,097).

^c^ Percentage of type of LIEHG by different modalities among all LIEHG.

### 3.2 Description of LIEHG

The LIEHG distribution varied significantly with age (from 24.9% at 25–29 years to 1.9% at 60 years and older, *p* < 0.001) (Table 3). The probability of diagnosing LIEHG in young women was 12.2% at 25–29 years, 14.1% at 30–34 years and 12.5% at 35–39 years. This probability decreased by half after 50 years: 5.2% at 50–54 years, 6.2% at 55–59 years and 5.5% at 60 years and older (Table 2). According to the number of previous screenings, we observed that few or no previous smears were significantly associated with a diagnosis of LIEHG (from 52.1% with no previous screening to 1.1% with five and more previous screenings, *p* < 0.001) (Table 3). Among the 1684 women without previous smears, 33.0% were under 30 years, and 29.2% were 40 years and older. In the study, 32.7% (505/1546) of LIEHG were diagnosed approximately 2 years (2 years ±6 months) after the previous screening smear, and this delay was significantly different between LIEHG and non‐LIEHG (*p* = 0.001, Table 3), followed by 22.4% (346/1546) and 21.7% (335/1546) at approximately 1 year ±6 months and 3 years ±6 months respectively. For the 819 women with one previous smear, the time to the next screening smear was less than 1.5 years in 21.5% (176 women) of cases. According to the Bethesda terminology, we observed that the type of smear was significantly associated with LIEHG detection (LSIL: 29.1%, HSIL: 27.2%, ASC‐US: 26.5%, ASC‐H: 14.9%, *p* = 0.0004, Table 3). The PPV of HSIL smears for the diagnosis of LIEHG was 70.3% (69.5%; 610/878 CIN 3, 27.3%; 240/878 CIN 2…) (Table 2). This PPV was 35.1% for ASC‐H smears (56.5%; 271/480 CIN 3, 42.3%; 203/480 CIN 2…), 22.5% for CIS smears, 17.2% for AIS smears, 9.7% for LSIL smears and 4.7% for ASC‐US smears (Table 2). According to age, atypical glandular cell smears and atypical endometrial cell smears were reported, respectively, on 189 and 240 women aged 45 years and older. These numbers were, respectively 79 and 220 cases in the group ‘Absence of histological indication’.

**TABLE 3:** Frequencies of high‐grade intraepithelial lesions in each characteristic.

Characteristics	LIEHG *N* (%)	NILM *N* (%)	Missing data	Absence of histological indication	Overall	*p* ^a^, ^b^
Overall	3230 (100)	6624 (100)	40 (100)	21,203 (100)	31,097 (100)	
Age						< 0.001^a^
25–29	804 (24.9)	1552 (23.4)	7 (17.5)	4222 (19.9)	6585 (21.2)	
30–34	714 (22.1)	1070 (16.2)	6 (15.0)	3285 (15.5)	5075 (16.3)	
35–39	586 (18.1)	1005 (15.2)	7 (17.5)	3104 (14.6)	4702 (15.1)	
40–44	494 (15.3)	986 (14.9)	7 (17.5)	3335 (15.7)	4822 (15.5)	
45–49	330 (10.2)	899 (13.6)	4 (10.0)	3215 (15.2)	4448 (14.3)	
50–54	150 (4.6)	564 (8.5)	4 (10.0)	2185 (10.3)	2903 (9.3)	
55–59	92 (2.9)	301 (4.5)	2 (5.0)	1085 (5.1)	1480 (4.8)	
60 and older	60 (1.9)	247 (3.7)	3 (7.5)	772 (3.7)	1082 (3.5)	
Number of previous screenings						< 0.001^a^
0	1684 (52.1)	3036 (45.8)	21 (52.5)	9729 (45.9)	14,470 (46.5)	
1	819 (25.4)	1695 (25.6)	6 (15.0)	5681 (26.8)	8201 (26.4)	
2	420 (13.0)	936 (14.1)	3 (7.5)	3042 (14.3)	4401 (14.2)	
3	185 (5.7)	517 (7.8)	7 (17.5)	1606 (7.6)	2315 (7.4)	
4	88 (2.7)	258 (3.9)	1 (2.5)	639 (3.0)	986 (3.2)	
5 and more	34 (1.1)	182 (2.8)	2 (5.0)	506 (2.4)	724 (2.3)	
Time taken to perform the positive test (months)—Around the year						0.001^a^
First screening	1684 (52.1)	3036 (45.8)	21 (52.5)	9729 (45.9)	14,470 (46.5)	
6 months and below	40 (1.3)	125 (1.9)	2 (5.0)	347 (1.6)	514 (1.7)	
1 year ±6 months	346 (10.7)	969 (14.6)	7 (17.5)	2697 (12.7)	4019 (12.9)	
2 years ±6 months	505 (15.6)	1087 (16.4)	7 (17.5)	3725 (17.6)	5324 (17.1)	
3 years ±6 months	335 (10.4)	682 (10.3)	2 (5.0)	2273 (10.7)	3292 (10.6)	
3.5 years and more	320 (9.9)	725 (11.0)	1 (2.5)	2432 (11.5)	3478 (11.2)	
Smear collectors						0.6^b^
General physicians	755 (23.6)	1485 (22.7)	8 (20.0)	5215 (25.1)	7463 (24.4)	
Gynaecologists	2337 (73.1)	4854 (74.3)	31 (77.5)	14,885 (71.7)	22,107 (72.4)	
Midwives	102 (3.2)	192 (2.9)	1 (2.5)	644 (3.1)	939 (3.1)	
Others	2 (0.1)	4 (0.1)	—	24 (0.1)	30 (0.1)	
Missing	34	89	—	435	558	
Types of positive smears						0.0004^b^
ASC‐US	855 (26.5)	2463 (37.2)	17 (42.5)	14,752 (69.6)	18,087 (58.2)	
LSIL	941 (29.1)	3039 (45.9)	16 (40.0)	5670 (26.7)	9666 (31.1)	
ASC‐H	480 (14.9)	613 (9.2)	3 (7.5)	270 (1.3)	1366 (4.4)	
HSIL	878 (27.2)	290 (4.4)	1 (2.5)	80 (0.4)	1249 (4.0)	
Squamous carcinoma in situ	9 (0.3)	30 (0.4)	—	1 (0.0)	40 (0.1)	
Squamous cell carcinoma	1 (0.0)	—	—	—	1 (0.0)	
Atypical glandular cells	65 (2.0)	147 (2.2)	3 (7.5)	162 (0.8)	377 (1.2)	
Atypical endometrial cells	—	24 (0.4)	—	266 (1.2)	290 (0.9)	
Adenocarcinoma	1 (0.0)	18 (0.3)	—	2 (0.0)	21 (0.1)	

Abbreviations: %, percentage distribution of LIEHG among variable's modalities; ASC‐H, atypical squamous cells cannot exclude HSIL; ASC‐US, atypical squamous cells of undetermined significance; HSIL, high‐grade squamous intraepithelial lesion; LIEHG, high‐grade intraepithelial lesions; LSIL, low‐grade squamous intraepithelial lesion; *N*, effective; NILM, negative for intraepithelial lesion or malignancy after histological investigation; *p*, *p*‐value, missing data and absence of histological indication have been excluded from *p*‐value estimation.

^a^ Pearson's chi‐squared test.

^b^ Fisher's exact test.

### 3.3 Factors Associated With LIEHG

The association between LIEHG and the age at positive smear, number of previous screenings, time taken to perform the positive test, smear collectors and types of positive results was investigated (Table 4). In multivariable analysis, LIEHG detection increased significantly with the age at positive smear [40–44: OR 1.56 (95% CI 1.23–1.97), 45–49: 1.77 (95% CI 1.39–2.27), 50–54: 2.37 (95% CI 1.75–3.22), 55–59: 2.28 (95% CI 1.59–3.31), 60 and more: 3.07 (95% CI 2.01–4.79)], depending on the type of positive smear [ASC‐H: OR 3.44 (95% CI 2.66–4.46), ASC‐US: 8.19 (95% CI 6.56–10.3), LSIL: 10.4 (95% CI 8.33–13.1), Atypical glandular cells: 5.80 (95% CI 3.79–9.02)] and with five or more previous screenings (OR 1.6 (95% CI 1.10–2.46)). This risk was reduced by 34% and 37% for test completion times, respectively, at 2 years ±6 months [OR 0.66 (95% CI 0.44–0.97)] and 3 years ±6 months [OR 0.63 (95% CI 0.42–0.95)] (Table 4).

**TABLE 4:** Analysis of the relationship (logistic regression model) between high‐grade intraepithelial lesions and predictive factors.

Characteristics	LIEHG *N* (%)	NILM *N* (%)	Risk analysis of LIEHG in a logistic regression model			
			Univariate analysis		Multivariate analysis	
Overall	3230 (100)	6624 (100)	Unadjusted OR (95% CI)	*p*	Adjusted OR (95% CI)	*p*
Age						
25–29	804 (24.9)	1552 (23.4)	RL			
30–34	714 (22.1)	1070 (16.2)	0.78 (0.68–0.88)	< 0.001	0.96 (0.77–1.20)	0.7
35–39	586 (18.1)	1005 (15.2)	0.89 (0.78–1.01)	0.081	1.14 (0.91–1.43)	0.2
40–44	494 (15.3)	986 (14.9)	1.03 (0.90–1.19)	0.6	1.56 (1.23–1.97)	< 0.001
45–49	330 (10.2)	899 (13.6)	1.41 (1.21–1.64)	< 0.001	1.77 (1.39–2.27)	< 0.001
50–54	150 (4.6)	564 (8.5)	1.95 (1.60–2.38)	< 0.001	2.37 (1.75–3.22)	< 0.001
55–59	92 (2.9)	301 (4.5)	1.69 (1.33–2.18)	< 0.001	2.28 (1.59–3.31)	< 0.001
60 and older	60 (1.9)	247 (3.7)	2.13 (1.60–2.89)	< 0.001	3.07 (2.01–4.79)	< 0.001
Number of previous screenings						
0^a^	1684 (52.1)	3036 (45.8)	RL			
1	819 (25.4)	1695 (25.6)	1.15 (1.04–1.27)	0.008	—	—
2	420 (13.0)	936 (14.1)	1.24 (1.09–1.41)	0.001	1.04 (0.89–1.22)	0.6
3	185 (5.7)	517 (7.8)	1.55 (1.30–1.86)	< 0.001	1.23 (1.00–1.51)	0.056
4	88 (2.7)	258 (3.9)	1.63 (1.27–2.10)	< 0.001	1.20 (0.90–1.60)	0.2
5 and more	34 (1.1)	182 (2.8)	2.97 (2.08–4.37)	< 0.001	1.62 (1.10–2.46)	0.019
Time taken to perform the positive test (months)—Around the year						
First screening^a^	1684 (52.1)	3036 (45.8)				
6 months and below	40 (1.3)	125 (1.9)	RL			
1 year ±6 months	346 (10.7)	969 (14.6)	0.90 (0.61–1.29)	0.6	0.84 (0.56–1.25)	0.4
2 years ±6 months	505 (15.6)	1087 (16.4)	0.69 (0.47–0.99)	0.049	0.66 (0.44–0.97)	0.041
3 years ±6 months	335 (10.4)	682 (10.3)	0.65 (0.44–0.94)	0.027	0.63 (0.42–0.95)	0.029
3.5 years and more	320 (9.9)	725 (11.0)	0.73 (0.49–1.05)	0.1	0.75 (0.49–1.12)	0.2
Smear collectors						
General physicians	755 (23.6)	1485 (22.7)	RL			
Gynaecologists	2337 (73.1)	4854 (74.3)	1.06 (0.95–1.17)	0.3	0.92 (0.78–1.08)	0.3
Midwives	102 (3.2)	192 (2.9)	0.96 (0.74–1.24)	0.7	1.06 (0.72–1.58)	0.8
Others	2 (0.1)	4 (0.1)	1.02 (0.20–7.35)	> 0.9	0.70 (0.06–15.4)	0.8
Missing	34	89				
Types of positive smears						
ASC‐US	855 (26.5)	2463 (37.2)	8.72 (7.49–10.2)	< 0.001	8.19 (6.56–10.3)	< 0.001
LSIL	941 (29.1)	3039 (45.9)	9.78 (8.41–11.4)	< 0.001	10.4 (8.33–13.1)	< 0.001
ASC‐H	480 (14.9)	613 (9.2)	3.87 (3.24–4.63)	< 0.001	3.44 (2.66–4.46)	< 0.001
HSIL	878 (27.2)	290 (4.4)	RL			
Squamous carcinoma in situ	9 (0.3)	30 (0.4)	10.1 (4.93–22.8)	< 0.001	—	—
Squamous cell carcinoma	1 (0.0)	—	—	—	—	—
Atypical glandular cells	65 (2.0)	147 (2.2)	6.85 (4.99–9.49)	< 0.001	5.80 (3.79–9.02)	< 0.001
Atypical endometrial cells	—	24 (0.4)	—	—	—	—
Adenocarcinoma	1 (0.0)	18 (0.3)	—	—	—	—

Abbreviations: %, percentage distribution of LIEHG among variable's modalities; 95% CI, 95% confidence interval; ASC‐H, atypical squamous cells cannot exclude HSIL; ASC‐US, atypical squamous cells of undetermined significance; HSIL, high‐grade squamous intraepithelial lesion; LIEHG, high‐grade intraepithelial lesions; LSIL, low‐grade squamous intraepithelial lesion; *N*, effective; NILM, negative for intraepithelial lesion or malignancy after histological investigation; OR, odds ratio; RL, reference level.

^a^ These two modalities correspond to the same women (1684 smears).

## 4 Discussion

This study estimated the prevalence of LIEHG among 732,203 cervico‐uterine‐smears. The prevalence of LIEHG was greater among young women. The probability of diagnosing LIEHG was twice as high in young women compared to those over 50. The prevalence of high‐grade squamous intraepithelial lesions was higher than that of high‐grade cervical glandular intraepithelial neoplasia. The findings revealed that HSIL smears had the highest PPV for diagnosing LIEHG and that adhering to the recommended screening deadlines reduced the risk of LIEHG. In the present study, the prevalence of LIEHG in the screened population was 0.4%, with a higher prevalence among women under 35 years. Similar outcomes were observed in previous studies [13, 24, 25, 26, 27]. In our study, this high prevalence of LIEHG in women under 35 years old can be explained by multifactorial causes, leading to a significant increase in risk factors for the occurrence of LIEHG in this age group:

- Smoking among young women: Grignon and Renaud [28] estimated the prevalence of smoking for a given cohort across 5‐year age groups. Their data show that for all cohorts of women, the prevalence of smoking was higher among younger women compared to older women. For example, for the 1946–1950 cohort, the smoking prevalence was 40% at ages 25–29 and 14% at ages 60–64. For the 1961–1965 cohort, the smoking prevalence was 56% at ages 20–24, 45% at ages 25–29 and 34% at ages 45–49 [28]. The estimated average daily cigarette consumption over a lifetime was also higher among younger women compared to older women. For example, women aged 30–34 in 2010 had smoked an average of 4.37 cigarettes per day since age 15, compared to 2.99 cigarettes for women aged 60–64 [29]. Previous studies have already shown that smoking is a risk factor for the persistence and even development of HPV‐dependent lesions [7, 8] so the high prevalence of smoking among young women could explain this high prevalence of LIEHG in women under 35 years old.
- HPV infection transmission: HPV infection is primarily transmitted through sexual contact, and risky sexual behaviours (early age at first intercourse, multiple sexual partners sexually transmitted diseases) [2, 3] among young people could also explain this high prevalence of LIEHG in women under 35 years old. Previous authors report that the prevalence of HPV infection peaks at around 20%–30% in women aged 20–24 and then decreases to around 3%–10% in those over 30 [30].

Numerous studies have demonstrated that widespread HPV vaccination of young boys and girls can significantly impact the incidence of LIEHG due to the high effectiveness of the HPV vaccine, reducing cervical HPV infections and decreasing the risk of recurrence after local excision treatment [4, 15, 16]. However, the HPV vaccine was introduced into the vaccination schedule in France in 2007. Although not mandatory, it is highly recommended for both girls and boys, typically starting at the age of 11 [31]. Therefore, we hypothesise that this introduction of the HPV vaccine has not had a protective effect on our study population. These findings should lead to particular vigilance in monitoring young women with a positive smear.

The France National Tobacco Control Program 2018–2022 reported a high prevalence of smokers among people with the lowest incomes in France: 33.6%, compared to 21.4% among those with the highest incomes, highlighting an aggravation of social inequality [32]. Similarly, previous studies have reported social inequality in cervical cancer screening related to age and social deprivation [33, 34]. This observation should prompt further studies to explore possible links between geographical characteristics (socio‐cultural context, lower adherence to prevention messages, social norms more favourable to smoking and less economic accessibility to treatment and care) and the incidence of LIEHG. The low prevalence of LIEHG lesions among women over 50 years could be explained by a decline in cervical cancer screening uptake after 50 years. In France, Hamers et al. reported a standardised coverage rate for triennial cervical cancer screening (2015–2017) of 65.5% at 25–29 years. However, this rate decreased significantly to approximately 53% for women aged 50 and dropped to 44.2% in those over 60 years.

The slight peak in the LIEHG prevalence among positive smears between 2013 and 2016 could be explained by the age‐based invitation waves (25–38 years in 2013, 39–50 years in 2014, 51–65 years in 2016) and the scaling up of recommendations to routinely perform an HPV test following an ASC‐US smear in 2013. In the experimental programme in Maine‐et‐Loire, Theurier et al. indicate an increase in the use of HPV testing as a reference control for ASC‐US from 5.3% in 2011 to 26.4% in 2014 [35].

As in some studies and in our experience, the prevalence of high‐grade squamous intraepithelial lesions was higher than that of high‐grade cervical glandular intraepithelial neoplasia [13, 36]. Previous studies reported that there are neither clinical nor colposcopic signs and no specific biomarkers to distinguish between atypical squamous and glandular cells; therefore, cytology is the main diagnostic tool [36, 37, 38]. In France, the 2020 recommendation to perform HPV testing as part of primary cervical cancer screening for women aged 30 to 65 years [17, 39, 40] could lead to a possible endometrial carcinoma being overlooked in an HPV‐negative patient over 45 years. Therefore, the bodies in charge of screening should carefully monitor and evaluate HPV status as a diagnostic indicator.

In this study, adherence to cytological screening recommendations by smear collectors may be questioned, with only 21.5% of women having their first two smears within an 18‐month interval. These results align with findings previously described by Tatar et al., which indicate that the most significant healthcare provider knowledge gaps are related to the higher sensitivity of the HPV test and age‐specific guideline recommendations for HPV testing [40]. Previous studies also indicated that entry into cervical cancer screening occurred from the age of 26, along with the screening invitation [41, 42]. These findings should prompt screening facilities to invest in ongoing training of healthcare providers and ensure the implementation of an appropriate invitation strategy concerning the age, results of previous screenings and follow‐up of women. In our study, the LIEHG risk was reduced by delays in carrying out the tests per the recommendations. Multivariate analysis shows an increased risk of LIEHG lesions associated with the completion of five or more previous smears. This unexpected finding, contrary to the literature [43], can be attributed to the performance of these smears (34 cases) outside the screening guidelines (50% of the smears were taken less than 18 months apart), with 38.2% of cases occurring in women under 40 years old (25–29 years: 2 cases, 30–34 years: 2 cases, and 35–39 years: 9 cases).

Previous studies reported that the diagnosis of CIN 1 has poor reproducibility and depends on rates of follow‐up after negative colposcopy [13, 37]. This significant finding should prompt the structures responsible for screening monitoring to allocate resources to ensure active follow‐up of LSIL or ASC‐US smears. Our findings revealed that following histological investigation, 29.1% and 26.5% of LIEHG, respectively, originate from LSIL and ASC‐US smears. We observed that the PPV of HSIL smears for the diagnosis of LIEHG was 70.3%; we could question the monitoring of the 290 cases of Negative for Intraepithelial Lesion or Malignancy after histological investigation and the 80 cases of absence of histological indication. Is this discordance due to a cytological/histological analysis error, a poor quality of sampling taking or the delay between the cytology and histology? With a PPV of 35.1% of ASC‐H smears for the diagnosis of LIEHG, the monitoring of these ASC‐H smears could be questioned. These results agree with previous data already emphasising the need to adapt clinical investigations to the identified precancer risk [5, 44]. Additionally, previous studies confirm the need to introduce and investigate new diagnostic tools for detecting LIEHG, including molecular tests for HR‐HPV DNA material, HPV mRNA transcripts or methylation assays [6, 45].

Some limitations need to be considered. We opted to focus on LIEHG detected in initial positive smears and did not analyse the diagnosis of LIEHG lesions during follow‐up. This methodological decision regarding the study population, coupled with the screening and diagnostic methods employed, resulted in a decreased number of LIEHG cases within the studied population. This may have posed challenges in comparing the prevalence of LIEHG lesions with other studies. The relevant data for screening evaluation within a population, such as HPV genotypes in positive smears and HPV vaccination status, were not included in this study because this information was not collected as part of the programme. However, this is a consequence inherent in retrospective studies that cannot question the results of this study. Additionally, the strengths of our study were the following: the quality of the database enables the identification and tracking of more than 3230 LIEHG, and the screening structure in Isère has been organising cervical cancer screening since 1990 [19] and has achieved a triennial coverage rate (2019–2021) estimated at 70.1% [46].

## 5 Conclusions

This study showed that the prevalence of LIEHG was higher among women under 35 years. To enhance early detection of LIEHG, we recommend strengthening screening among young women as well as the active follow‐up of low‐grade smears (LSIL or ASC‐US). Additionally, screening facilities should allocate resources to developing interventions targeting vulnerable women. We observed a low adherence to cytological screening guidelines by smear collectors, which prompts us to recommend that the screening facilities ensure proper training for smear collectors, especially considering that the choice of screening test will depend on the woman's age.

## Author Contributions

**Christian Balamou:** conceptualization (lead), data curation (equal), formal analysis (equal), methodology (equal), project administration (lead), visualization (equal), writing – original draft (lead), writing – review and editing (lead). **Karine Zysman:** data curation (lead), formal analysis (equal), methodology (equal). **Cécile Olicard:** data curation (equal), formal analysis (equal), methodology (equal), software (lead). **Anne Garnier:** data curation (equal), formal analysis (equal), methodology (equal). **Arnaud Seigneurin:** conceptualization (equal), formal analysis (equal), methodology (equal), supervision (lead), validation (lead), writing – review and editing (equal).

## Ethics Statement

Before analysis, all data were anonymised. The screening database had a favourable opinion from the institution that oversees the ethics of data collection (‘Commission Nationale de l'Informatique et des Libertés’). According to current French legislation, a study analysing existing databases does not require approval from the Ethics Committee of clinical research centres [47].

## Conflicts of Interest

The authors declare no conflicts of interest.

## Data Availability

Data are available on request due to privacy/ethical restrictions.
